# ‘Elastic band strategy’: women's lived experiences of coping with domestic violence in rural Indonesia

**DOI:** 10.3402/gha.v6i0.18894

**Published:** 2013-01-02

**Authors:** Elli Nur Hayati, Malin Eriksson, Mohammad Hakimi, Ulf Högberg, Maria Emmelin

**Affiliations:** 1 Faculty of Psychology, Ahmad Dahlan University, Yogyakarta, Indonesia; 2 Department of Public Health and Clinical Medicine, Epidemiology and Global Health, Umeå University, Umeå, Sweden; 3 Center for Health and Nutrition Research Laboratory, Gadjah Mada University, Yogyakarta, Indonesia; 4 Women's and Children's Health, Uppsala University, Uppsala, Sweden; 5 Department of Clinical Sciences, Social Medicine and Global Health, Lund University, Lund, Sweden

**Keywords:** domestic violence, coping, lived experience, Indonesia

## Abstract

**Background:**

Experiencing domestic violence is considered a chronic and stressful life event. A theoretical framework of coping strategies can be used to understand how women deal with domestic violence. Traditional values strongly influenced by religious teachings that interpret men as the leaders of women play an important role in the lives of Javanese women, where women are obliged to obey their husbands. Little is known about how sociocultural and psychosocial contexts influence the ways in which women cope with domestic violence.

**Objective:**

Our study aimed to deepen our understanding of how rural Javanese women cope with domestic violence. Our objective was to explore how the sociocultural context influences coping dynamics of women survivors of domestic violence in rural Purworejo.

**Design:**

A phenomenological approach was used to transform lived experiences into textual expressions of the coping dynamics of women survivors of domestic violence.

**Results:**

Experiencing chronic violence ruined the women's personal lives because of the associated physical, mental, psychosocial, and financial impairments. These chronic stressors led women to access external and internal resources to form coping strategies. Both external and internal factors prompted conflicting impulses to seek support, that is, to escape versus remain in the relationship. This strong tension led to a coping strategy that implied a long-term process of moving between actively opposing the violence and surrendering or tolerating the situation, resembling an elastic band that stretches in and out.

**Conclusions:**

Women survivors in Purworejo face a lack of institutional support and tend to have traditional beliefs that hamper their potential to stop the abuse. Although the women in this study were educated and economically independent, they still had difficulty mobilizing internal and external support to end the abuse, partly due to internalized gender norms.

In the early 1990s, violence against women became a focus of international attention and concern (1). It is now considered a major social and public health problem, as well as a human rights issue, in which governments have the right and obligation to intervene (2). Furthermore, the World Health Organization (WHO) as well as studies from other sites has concluded that domestic violence against women is a serious cause of physical and mental health impairment (3–7). The WHO multicounty study (4) not only confirmed the seriousness of domestic violence worldwide but also showed that the prevalence varied between countries. Lifetime and current physical violence ranged between 13–49% and 3–29%, respectively, whereas lifetime and current prevalence of sexual violence ranged between 6–50% and 1–29%, respectively. Studies around the globe have shown that multiple factors put women at risk of domestic violence, including the woman's and her partner's past history of violence, their current demographic, socioeconomic, and cultural situation, as well as their individual behavioral and relationship characteristics (4, 6, 8, 9).

Domestic violence against women refers to abusive acts that consist of three elements: boundaries of the relationship between the perpetrator and the abused, the norms of acceptable behavior, and the specific acts that constitute violence as the manifestation of subordination (10). In different cultural settings, women live in a wide variety of family structure arrangements. The violence that occurs is not limited to physical harm and may also be perpetrated by family members within the household other than the intimate partner. This definition acknowledges multiple explanations for the violence and could lead to more inclusive interventions that accommodate the experiences of all women (10).

Experiencing an abusive marriage is a complex phenomenon and is considered a chronic and stressful life event (11). Many studies have focused on identifying external factors that influence a woman's decision to leave or to stay in an abusive marriage (12–16). Others have emphasized the internal factors of female survivors (17–21) or focused on both external and internal factors (16, 21–24). However, the reasons why women decide to stay are less understood. A theoretical framework of coping strategies can be used to better understand how women manage the chronic stress associated with abuse. Coping is a cognitive and behavioral effort undertaken to manage the external and/or internal demands of a taxing situation (25). Thus, coping is a mechanism of overcoming a stressor, and this mechanism cannot be separated from what is called the coping resources because those resources directly determine the coping responses (21).

There are three important pairs of coping strategies described in the literature. The first is *problem-focused* and *emotion-focused* coping (25), the second is *approach* and *avoidance* coping (26) and the third is *engagement* and *disengagement* coping (27). The avoidance coping response is characterized by cognitive avoidance, resignation/acceptance, seeking alternative rewards, and emotional discharge (26). The approach coping response is characterized by logical analysis, positive reappraisal, seeking guidance/support, and problem solving. According to Tobin et al. (27), the concept of engagement/disengagement coping is a combination of the coping approaches proposed by Lazarus and Folkman (problem focused and emotional focused), where engagement coping includes problem solving, cognitive restructuring efforts, emotional expression, and social support, whereas disengagement includes problem avoidance, wishful thinking, self-criticism, and social withdrawal (27).

## Social context of women in Indonesia

The ideology of family harmony has been set as a priority by the Government of Indonesia, and women have been given a significant role in maintaining that harmony (28). Legally, the Marriage Law No. 1/1974 states that a husband and a wife have equal rights within the marital relationship, although husbands are stated to be the head of the household and wives are responsible for the household. Only in the last decade, under the new political reformation government was violence against women officially declared as a national problem. A Domestic Violence Act was endorsed in Indonesia in 2004. However, a national study conducted by Rifka Annisa (a non-government organization) in 2008 revealed that this Act had not been satisfactorily implemented and therefore not well understood by ordinary people (29).

## Domestic violence against Javanese women

Java is the main island of Indonesia, and Jakarta, the capital city of Indonesia, is located on this island. In Javanese tradition, women are constrained by the traditional feminine ideal that extols the virtues of submission and obedience. Javanese traditional values are strongly influenced by Islamic teachings that interpret men as the leaders of women and therefore require a woman to be obedient to her husband. Once married, a woman is bound to fulfill the socially prescribed roles of housekeeping, childbearing, and supporting her husband. The ideology of harmony is widely applied as a marriage norm and is referred to as *njaga praja*, meaning that the husband's honor must be protected from people outside the family (30). This spirit of harmony implies that conflicts and oppression within a family should not be discussed. Therefore, domestic violence often is hushed up, as acknowledging it would reveal a lack of harmony within both the family and the nation (31).

In a population-based study, using the WHO multicountry questionnaire, we found that the lifetime prevalence of physical and sexual violence among rural Javanese women in Purworejo District is 11 and 22%, respectively (5, 32). Of the women who had been physically abused, nearly 50% had told nobody about their experience of violence, while 33% had confided in their parents. The identified risk factors for experiencing lifetime violence included husband's age (>35 years), education (>9 years), and husband's psychosocial behavior, such as being unfaithful, drinking alcohol, fighting with other men, and having witnessed domestic violence as a child. From the women's side, being economically independent and having traditional views on gender norms were risk factors for domestic violence (32). However, little is known about how sociocultural and psychosocial contexts influence the interpretation of the meaning of abuse by the rural women of Purworejo and how that interpretation may influence their decision to take action. In this study, we aimed to deepen the understanding of how rural Javanese women cope with domestic violence, with a particular focus on violence perpetrated by the intimate partner. Focusing on rural women is important because they often have limited access to services, lower education, and more often live under socioeconomic constraints (33, 34). We hope that the study findings will enable policy improvements to increase the quality and accessibility of services for battered women in (rural) Indonesia in the future. Specifically, the aims of our study were to understand how the sociocultural context influences the coping dynamics of female survivors of domestic violence in rural Purworejo and to discuss the policy implications for care and prevention.

## Methods

### Study setting

We conducted the study in Purworejo District, Central Java Province, which is located 60 km west of Yogyakarta Province. According to the 2010 census (35), Purworejo District had a population of 695,427, with a total area of 1,035 km^2^, including coastal, lowland, highland, and hilly areas. Although urban centers are found in the district, 85% of the population lives in rural areas, with farming being the major occupation.

In 2000, the district government formally appointed the Office of Social Affairs and People Empowerment (OSAPE) to form a task force unit with the main function of providing a complaints desk for women survivors of domestic violence. Because of resource limitations, they only began documenting their activities in 2005. By the end of 2007, they had documented 27 domestic violence reports from women in the district.

### Study design

This study is based on a phenomenological approach, which is suitable for transforming lived experience into a textual expression of its essence (36). Phenomenology provides women survivors of domestic violence an opportunity to voice their own perspective of their life experiences (21). Within an empathetic interview, a woman survivor of violence is able to formulate her life history from the past to the present, and can explore, share, and validate her feelings and insights (21). Giving women survivors an opportunity to express their internal struggle in the face of abuse provides a valuable depiction of the development and use of inner resources that facilitate survival, strength, identity formation, and protection (20).

### Data collection

In-depth interviews were conducted with women survivors of domestic violence. The sampling technique used was criterion sampling, by which individuals who had been exposed to domestic violence and were willing and able to articulate their experiences were approached (37). In this study, we received information about possible informants (women survivors) from OSAPE. Only limited OSAPE staff members were available, one part-time and three full-time employees. In addition to other responsibilities, they were involved in counseling, referral, and outreach activities related to domestic violence. From the office, we were introduced to nine women who had been in contact with them for assistance. These women were all invited to participate in the study. After being informed of the research aims, seven women consented to be interviewed. The two that declined to be interviewed were not ready to discuss their domestic violence experience. All interviews were performed between December 2006 and August 2007, in places that the informants felt comfortable talking about their experiences. Two interviews took place at OSAPE, which some of the women were already familiar with. The other five interviews were conducted in other offices or in the informant's home. The interviews took between 1 and 1.5 hours, with only the woman survivor and the researcher present.

A semi-structured interview guide was developed and used during the interviews. The interview focused on the women's marriage history, the abuse experiences, efforts made to overcome the problems, social interactions, reactions and responses, the children's situation, and the women's reasoning for deciding to stay or to leave the marriage.

### Data analysis

The analysis followed a phenomenological guide developed by van Manen that combines features of descriptive and interpretative phenomenology (36, 38). According to van Manen, phenomenological inquiry methods cannot be formalized into a series of technical procedures; however, a variety of activities may fall into two types: empirical and reflective methods. Empirical inquiry activities aim to explore the range and varieties of pre-reflective experiential material that is appropriate for the phenomenon under study (39). Reflective inquiry activities aim to interpret the aspects of meaning or meaningfulness that are associated with this phenomenon. In this study, after reading the transcripts several times, we got a ‘sense’ of the data and a robust image was achieved. In the next step, we coded phrases, sentences, or statements that were significant for the purpose of this study (meaning units) and grasped the meaning of the identified meaning units by re-formulating them (descriptive level). Entering the reflective part of the analysis, we interpreted the descriptive formulated meanings and identified the subthemes (interpretative level). Finally, we clustered the subthemes into themes and integrated them into a comprehensive phenomenon (see Table 1).

**Table 1:** Data analysis structure (adapted from van Manen [34, 35])

Themes Sub themes	Reflective inquiry activities	Interpretative level
Formulated meaning unit		
Meaning unit	Empirical inquiry activities	Descriptive level
Transcript		
Interview with the respondent		

Table 2 below illustrates the analysis process and gives an example of how the subthemes emerged from the text.

**Table 2:** Data analysis examples

Empirical inquiry		Reflective inquiry
	
Meaning unit (from the transcripts)	Formulated meaning (descriptive analysis)	Subtheme (interpretative analysis)
I wore a Muslim veil because he wanted me to. I had to keep everything covered. I wasn't allowed to look beautiful and I wasn't allowed to get dressed up. As a wife I did what he wanted. If he told me what to do, I did what he said … the important thing to me was to maintain peace at home …	He directed her performance. She was obedient and complied with his orders to keep the marriage intact.	Being controlled on how to perform. Obedience and submissiveness as strategy to maintain the family harmony. Traditional belief about women's role in marriage.
		
I couldn't take it anymore so I told the head of the local Health Center when he asked, ‘Why are you always getting chicken pox?’ that actually they were really cigarette burns, not chicken pox.	She confessed during health attendance that she was burnt by his cigarette butts.	Self-disclosure during health attendance. Physically tortured.

### Ethical considerations

Ethical approval was obtained from the ethical review board at the Faculty of Medicine, Gadjah Mada University in Yogyakarta, and from the Purworejo District Government. Written consent was obtained from all participants prior to the interview. To protect confidentiality we used the codes S1–S7 to name our informants.

## Results

The seven women who consented to participate in the study had an average age of 39 years. Five of them had finished their bachelor's degree, while two had finished high school. The duration of marriage was 9–22 years and all had at least two children together with their abusive husband. Five women worked as civil servants at the government bodies in Purworejo, while two ran small shops (*warung*). Three women were divorced prior to the interview, while the other four were still living in an abusive marriage (see Table 3).

**Table 3:** Profile of the informants

Identity code	Age	Education	Occupation	Number of children	Types of violence experienced
S1 (divorced)	35 years	Bachelor degree	Primary health care staff	2	Physical, sexual, emotional & economic
S2	35 years	High school	Run a small shop at home	2	Physical, emotional & economic
S3 (divorced)	33 years	High school	Run a small shop at home	2	Physical, sexual, emotional & economic
S4	50 years	Bachelor degree	Civil servant (health district office)	2	Physical, emotional & economic
S5	43 years	Bachelor degree	Civil servant (teacher)	3	Physical, emotional & economic
S6	42 years	Bachelor degree	Civil servant (teacher)	3	Physical, sexual, emotional & economic
S7 (divorced)	38 years	Bachelor degree	Civil servant (sub district office)	2	Emotional & economic

Three women (S1, S3, and S6) suffered from all four forms of abuse (physical, sexual, emotional, and economic), three women (S2, S4, and S5) suffered from three forms of abuse (physical, emotional, and economic), and one women (S7) suffered from two forms of abuse (emotional and economic). The extent of abuse faced by these women was moderate to severe, with one woman suffering from severe injuries (S1).

Several of these women expressed feelings of being *financially abandoned*. S1 and S3 explained how they struggled to survive without enough economic support from their husbands and described the humiliating feelings this caused:

On about the 4th of March after the police force payday he came home after having disappeared for some days. He threw me three hundred rupiahs in front of my older sibling. I told him it wasn't enough … I asked for one hundred more. In front of the kids he said, “Go sell yourself … you should try it.” (S1)

S2, S4, S5, S6, and S7 also expressed their feelings about a strained financial situation, where they had to manage themselves to solve the financial needs within the household because of their husbands’ unfairness in sharing their income. Experiencing their husband stealing their savings or leaving no cash in the house for the family's daily needs were common problems for these women.

The women were exposed to various forms of *physical abuse* that caused minor as well as major injuries. S1 and S2 received major injuries when they were beaten, causing bleeding and fainting, while S3, S4, and S5 experienced mild to moderate physical abuse:

… every time he noticed me … put makeup on my face … when I prepared myself for the office, he made cynical comments … once he tried to remove the face powder from my face … he pushed me backward and roughly wiped a towel over my face to mess up my make-up … he did that because he was jealous, thinking I will have an affair with another man. (S5)

Jealousy caused physical abuse for S4, while S1 and S6 experienced physical abuse in addition to *sexual abuse*:

One night he woke me up and asked for sex … I woke up but was not ready to suddenly have sex with him … I mean … I need some time to prepare myself to have sex with him … but then he beat me up! It was just after midnight. His beating really made me awake, so I ran away from him and went into my kid's room. He said that wherever I run, I'm still his wife, so he might do anything to me! He did it several times … beat me … because of my refusal to his sexual requests. (S6)

Overall, the informants described a poor life situation and feelings of being destroyed by the abuse they experienced by their husbands.

Our analysis resulted in four main themes (italics) and six subthemes (bolded) that together illustrate the coping dynamics of these women survivors. First, the *ruined self* refers to the negative effects of domestic violence, that is, the overall loss of self-respect and confidence that the women had in common, even if they handled the situation in different ways. Second, the *inner realities* illustrate the dilemma the women felt when choosing between **fight to rescue** or **maintain the harmony**. Third, the *outer realities* show the influence of significant others and more formal institutions when functioning as **supporting actors** or **closed gates**. Fourth, the *elastic band strategy* demonstrates a coping strategy characterized by movement back and forth between **opposing** the violence or **surrendering** and accepting the situation. Below is a more detailed presentation of the results under the theme and the subtheme headings. Quotations from the interviews are given to illustrate how the informants own words have guided the interpretations.

### The ruined self

This theme describes the immediate effects as well as the long-term impacts of domestic violence on the person. The theme differs from the others in that it does not independently and on its own relate to coping. However, it describes the negative consequences of experiencing domestic violence, which thus creates the need to cope. Without ‘the ruined self’ there would be no need to cope. The women expressed a situation where they were totally disrespected, degraded, belittled, abandoned, and subordinated as human beings. Beatings, assaults, scolding, yelling, financial abandonment, hampers and control were overt expressions of their husbands’ power over them as wives. Because of this chronic abuse, these women suffered from immediate injuries as well as long-term negative impacts on their self-integrity. S1, S4, S5, and S6 described how they were accused by their husbands of having affairs with other men. This accusation had an almost fatal effect on S1's life, as that accusation was related to her husband's attempt to force her to sign divorce papers. As a result, S1 attempted to commit suicide:

Early in the morning, I was taken to hospital for two weeks. I was unconscious and on medication for a week. I took sleeping pills and poison used to exterminate pets. I felt like there was no way out. (S1)

Another of the informants, S4, developed gynecological problems because of her husband's adultery with a prostitute:

Last time I developed problems with my genitalia … painful and itchy like eczema down there … I showed it to him. I said, “You see, here is the bad thing again, from you! This must be caused by you and your prostitute!” (S4)

S5 never experienced any physical or sexual violence, but she suffered from emotional and economic abuse. However, she experienced physical symptoms because of her anger and frustration. Living in a marriage with chronic conflict had obvious effects on both her physical and mental health:

My only complaint is deep regret here (pointing her chest) … at the beginning I developed high blood pressure, and often got headaches. (S5)

S1, S3, and S4 expressed their concern about their children because they were also exposed to their father's abusive behavior toward their mother:

My eldest child was shocked to see me being beaten and bleeding. He immediately got a nosebleed … he was stressed. (S1)

Being a victim of domestic violence also caused negative psychosocial effects on these women's interactions with others. S6 lost her reputation after being slandered by her husband:

He accused me of having an extramarital affair, using my money to have fun with my extended family, and created a negative rumor about me among my colleagues. He directly came to the school principal and raised negative speculations that ruined my reputation. He said, “If something bad happened in the future at this school, don't blame me as I've already warned you about her.” So the next day the principal called me and cancelled my promotion for the master program. (S6)

The descriptions above clearly illustrate that domestic violence had a devastating impact on the women's physical and mental health, as well as their economic and psychosocial situation. Thus, experiences of ‘the ruined self’ led to a need to cope with this chronic stress.

### The outer realities: ‘supporting actors’ versus ‘the closed gates’

This theme and the subthemes describe how the informants accessed their outer resources to form their coping strategy. Apparently, these women oscillated between getting appropriate support and being denied either social or institutional support from their networks. Mostly, support came from family members and friends, but at times these social networks also behaved as ‘the closed gates’ because they did not give any sanction to the batterer. The formal actors, either affiliated to government or nongovernment organizations, also played a significant role, because women who had already successfully terminated their abusive marriage received institutional support, while those who were still in the relationship received nothing.

My big family, my father, mother, brothers and sisters were all on my side. (S7)

We were so poor that time, so my father kept sending me a monthly stipend because he was so worried. Life was very hard at that time. My husband always spent the stipend from my father. He tried to do a lot with the money, but nothing that he tried succeeded. (S4)

All of the children of the women in our study had witnessed their mother being abused by their father in various ways. Because they knew how their mother had been acting within the household, the children were on her side:

The older one … she shouted loudly at him one day as he treated me bad, she said to him, “What else do you want from Mama? What is her fault? Are you crazy or what?” He was so upset with her comment and yelled at her, calling her a sinful daughter for being that rough with her own father, “God will not grant you prosperity,” he said. She replied to him, “Prayers from a father such as you will be rejected by God.” (S4)

Besides support from significant others surrounding the women, S1 had received support from her husband's work place (employer). Her bravery in reporting her situation was followed up with her husband's employer intervening in her domestic violence problems:

I went to the head of the operational section, who was third in charge at the District Police Station. I said, “Sir, please help me … my husband is living with another woman.” He said, “Are you sure you are ready for this?” I said, “I'm ready … if possible sir, do something now … don't wait until tomorrow.” They immediately went in a car to arrest him. (S1)

S1 received a good response from her husband's institution. On the contrary, even though she often complained to OSAPE, she felt that this office could not really fight for her rights. S3 was pursuing her legal process in another city around 100 km north of Purworejo, where there was a nongovernment organization that had specific services for women survivors. In the end, she received tangible legal support and won her case. S7 was supported by her husband's office, which was committed to act because of his violation of the civil servants’ rules, that is, he married a second wife without consent from the first one.

However, some women informants were not defended by their immediate surroundings. S1 was in a big quarrel with her husband that ended in a fight, in which she was kicked and stomped in the abdomen and then expelled from the house along with her children. She asked one of the neighbors to mediate between them, but he refused to do anything to support her:

We slept at a mosque for three nights. Not even one person from my house helped us, not even one in the name of God. They saw me taking my things so they knew. I took clothes and a bag from the house. In Surorejo Village … no one helped me. (S1)

S2 was hit and kicked inside the house, and she screamed for help. Her mother came to help, while the neighbors did not do anything:

He hit and kicked me so hard that I collapsed … then he banged the door and went out … I screamed and my kid cried a lot … so my mother came and helped me, and the neighbors came over and asked my husband what had happened but he kept silent. And then he went to his uncle's house … just like that. (S2)

S6 received some material and financial support from her family, but no one saved her from her husband's beatings. Meanwhile, S5 complained to her mother-in-law about her husband's lack of contribution in financing the household, but her mother-in-law neglected her:

He had a job and projects, but he did not share his earnings for running the household. And when I complained to my mother-in-law, she said, “Why should you complain, don't you have your own job? Can't you can manage your life with the salary that you get?” (S5)

These descriptions illustrate that the outer realities, either individual or institutional, consisted of supportive actors as well as closed gates. The most reliable support came from family, while neighbors were less supportive and even tended to be ignorant toward domestic violence in the neighborhood. Concerning institutional responses, the experiences of S1, S3, and S7 of receiving institutional support showed the significant impact that this type of support could have. For these women, the institutional support gave them the strength to cope, and became a turning point, leading to the decision to leave their abusive husbands.

### The inner realities: fight to rescue versus maintain the harmony

This theme illustrates the internal/inner realities that influenced how the women coped with the domestic violence they experienced. On the one hand, their internal resources reflected a powerful self that was capable of fighting and rescuing themselves and their children. On the other hand, their inner resources also reflected a powerless self, influenced by internalized norms that require them to be a good wife and mother by maintaining the family harmony. A powerful self among these women was shown in their assertiveness and self-authority over their daily life:

The thing that drove me the most to divorce was that I wanted it to end it rather than continuing to live a life like that. I felt worried about the children, and secondly, I didn't want to live as a woman who is constantly being put down and belittled … I wanted the divorce. I wasn't afraid of the future. (S3)

Assertiveness was a quality that was possessed by all women in this study, and it was expressed differently according to personal style. This is actually a good modality for women to raise their voice within an intimate relationship.

Another quality that reflected a powerful self among these women was good self-regulation, which indicates a personal maturation among them:

One night he came home very late, and the next morning I still talked to him in a very pleasant manner … although he answered in a rough way … I talked to him politely, asked what he needed and wanted … I did that to protect the kids from imitating their father's rough behavior. (S7)

Another quality of these women that reflected a powerful self was self-confidence in maintaining their work performance outside the house. As we know, five out of seven women in this study were working at government bodies, and they all managed to perform their work well:

He is a civil servant just like me, but I think I had a better career than him at the time. I did many papers for different presentations, and got many points for that, while he didn't. So, actually I was more advanced than him in terms of career. I am a very independent person. (S5)

These women had good self-confidence, which could have functioned as an inner resource for managing domestic abuse. However, experiencing domestic abuse had weakened this powerful self, in favor of the more powerless self. The significance of the powerless self among these women was reflected in their beliefs about how to behave as a good wife in maintaining and keeping their marriage intact. Because of the internalized norms about being a good wife, they almost had no self-authority over their life:

“Why should you live like this? Why should you?” my father asked, and he asked me to move to his town several times, but it's so strange that I did not dare to do so … so strange that I always confirmed his (husband's) words, did what he asked, and had no bravery to act against him. (S4)

S5 and S6 expressed their powerlessness by waiting and asking their abusive husband to initiate a separation, which obviously would never happen:

That is what I feel … with everything he's done to me … he hurt me, betrayed me … I would very much indeed want to be away from him … divorce … but it's supposed to be his initiative … not mine. (S6)

S2 even denied that arguing and hitting are bad in a marriage, and therefore she did not really mind what her husband did to her:

Yeah … he did this to my head (pushing her head with her hand) … but it didn't really hurt. He always did that to my head. He always did it to the kids too. I think that it's just normal arguing between husband and wife. I think arguing is normal. (S2)

The next aspect that became an internal struggle to cope with domestic violence in this study was their concern about the children. S2 and S5 shared the experience of thinking about separation, but every time they started thinking about their children, they felt a deep concern about them:

That is exactly what is in my mind … if I leave him, I may be free and become a free woman … but what about my kids? (S5)

S1 and S6 were convinced that domestic violence had bad consequences for their children, and that is why they were concerned about how to protect their children from further exposure to the marital abuse:

At night I get scared that he's going to come and take the kids … I go and check my kids and just hold them both … I say, “Oh they are still here!” They are the most valuable thing to me. Let me be poor, but I don't want to lose my kids … I was stupefied by their father. I don't want that again … don't want him to meet with the little ones … he's not allowed to. (S1)

The above quotes describe how concern for their children pushed them from one side to another. Their concern for their children apparently strengthened them to fight and rescue both themselves and their children. However, at the same time, it weakened their inner power because of the internalized norms about their responsibility to maintain family harmony.

### The ‘elastic band strategy’: opposing the violence versus surrender and stay

This theme describes how the women in this setting coped with domestic violence. Analyzing their stories showed a situation whereby the women seemed to cope using what we have labeled the ‘elastic band strategy.’ This strategy implied a constant stretching, by making efforts to oppose the violence, for example, through spiritual framing, seeking outside support, being assertive, and trying to make a positive diversion. However, the stretching was often followed by withdrawal and surrender through submissiveness, keeping silent, or ignoring their husband's behavior.

An example of how women coped by being submissive is illustrated below:

I wore a Muslim veil because he wanted me to. I had to keep everything covered. I wasn't allowed to look beautiful and I wasn't allowed to get dressed up. As a wife, I did what he wanted. If he told me what to do, I did what he said … the important thing to me was to maintain peace at home. (S1)

S2 was also abused by her in-laws, as she lived in the same house as them. For her, being submissive was the most effective way to prevent further conflict with her husband and in-laws. A submissive act was usually followed by an act of silence, as expressed by S5:

My youngest kid saw it not so long ago … she said, “Mom, what happened?” I said to her, “He (husband) just broke down a door again.” But my neighbor never heard anything, because I always kept quiet about whatever he was mad about in our home. (S5)

Most of these women had noticed some strange behavior by their husband at an early stage of their marriage, but had ignored it:

I moved to another town with the kids, he was supposed to would follow later on. One day, I telephoned his neighbors and asked how he was. The neighbor said, “He does not live in the house any more … Oh, you didn't know that? He's living somewhere else now with another woman. You need to come here!” But I closed my eyes and ears. I thought, yeah … maybe it could happen if he was far from his wife, but I was sure that he wouldn't be living with another woman. I didn't believe it. (S1)

All informants described how they had ignored their husband's violent tendencies at the beginning of their marriage, but later they became aware that this did not improve their situation. These women used different methods to oppose their abusive husbands and thereby end the abuse.

Two women, S1 and S4, described how they framed their life in a spiritual way, something that is commonly practiced by people in this area:

I said that God never sleeps … I prayed … during the night, I just prayed, asking God to help me, to bring the truth to light. (S1)

Some of the women in this study undertook the brave action of initiating a report of their husband to the authorities. S1, S3, and S4 reported their husband's beatings to the police:

It was painful when he pushed me … my belly got hurt … I made a report to the police right away. (S3)

Other than making a legal report, some of the women were also active in seeking additional financial support:

I asked for assistance from the Office of Social Welfare and got some money to start a small business. Otherwise, where would the money come from for telephone, electricity and water? (S3)

Another way for these women to cope was to disclose the abuse to family or friends. All women in this study, except S5, eventually disclosed their abusive experience to family or friends:

Finally, after 10 days of keeping the secret to myself, I could not bear it any longer and I cried to my friend. (S7)

S5 undertook another brave action. She felt so frustrated by her husband's violent behavior, and she thought about how she could achieve a better future life by executing a positive diversion:

I became more and more frustrated to see what he did over and over again … and then I started to think about how to change my life into a happier one … I started to prepare myself for higher education, so that in the long run I will have a better income and a stable career. (S5)

Overall, the coping dynamics among the women in this study showed a complex pattern where the outer and inner realities could simultaneously act as factors that facilitate fighting against the violence and also as factors making the women surrender and tolerate the violence.

**Fig. 1:**
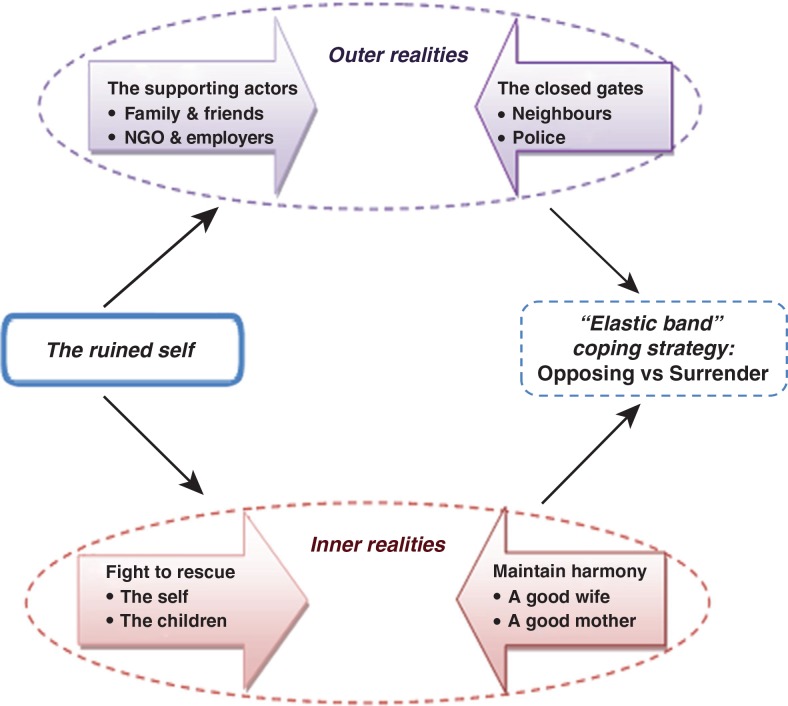
Coping dynamics of women survivors of domestic violence in rural Purworejo.

Figure 1 depicts how the women's experiences of chronic abuse (stressors) have ruined their personal life because of the associated physical, mental, psychosocial, and financial impairment. These chronic stressors led the women to evaluate and access their external and internal resources to form coping strategies. Apparently, both external and internal resources contained conflicting realities. Within their external resources, they found relatively good support from friends and family members, and from their husband's employers. However, at the same time, they received less support from their neighbors or the police. From their internal resources, these women also experienced a conflicting situation. They felt a need and a responsibility to rescue themselves and their children, which conflicted with the internalized norm of their role in maintaining family harmony by being an obedient wife and a good mother. This strong tension led to the *elastic band strategy*, which implies a long-term process of moving between actively opposing the violence and surrendering and abiding the situation a bit longer, before possibly, eventually leaving the relationship.

## Discussion

All women in this study suffered from moderate to severe forms of abuse from their partners, which resulted in a decision to either terminate the marriage through divorce or stay in the relationship but struggle to cope with the abuse. The overall strategy was described as *an elastic band* that stretches in and out to adjust to the immediate needs related to the violent episodes. The informants’ lived experience shows that coping behavior is a result of a complex interaction between the availability of inner and outer resources that in turn guide their decision to stay or to leave the marriage.

This study shows that the women exposed to violence felt that they received *appropriate social support from family or friends* when they asked for it but *lacked support from neighbors*. The importance of this support has been emphasized by others (14, 21, 40). Even if it often only temporarily slows the abuse, social support is viewed as important for improving the coping capacity of women survivors of partner abuse (40). Having a trusted colleague available to discuss one's problems with has also been shown to be beneficial (41). Other studies have found that battered women tend to have low levels of perceived social support (22, 40). This might be because of the common norm of viewing domestic violence as a private issue in which outsiders should not intervene. In our study setting, traditional values, shared by most Javanese, state that once a woman marries a man, she belongs to him, and the parents no longer have power over her. Our previous findings (5) have shown that the majority of women in this study setting (94%) agreed to the statement ‘Family problems should not be discussed with people outside the family.’ On the other hand, more than half of the women (59%) agreed that in the case of violence, ‘Others outside the family should intervene.’

The women in this study lacked proper *institutional support* from the Police Department, legal institutions, or other related government bodies. Only one of the women who had reported their husband to the police was followed up seriously. However, the women who decided to leave their abusive husbands felt that they received tangible support from the government bodies that their husbands were affiliated to or from human rights organizations (nongovernment organizations). Receiving positive responses from organizational bodies gave these women confidence and a feeling of being able to change their situation. This means that support from formal institutions may significantly contribute to encouraging women who have decided to end an abusive marriage. This is in line with the framework proposed by Liang et al. (14) about the leaving process among survivors of intimate partner violence, and findings from the study of Koepsell et al. (15), where women who left the relationship were significantly more likely to have successfully accessed services from domestic violence agencies or other public or private assistance, compared with those that had not left the relationship.

Internally, women fought against the internalized norm that a woman's role is about being a good wife and a good mother (42). Thus, in a society that places the burden of family harmony on the woman, a failed marriage must be her fault. This suggests that a commitment to the relationship may be a significant factor in the decision to tolerate abuse (43). In this study setting, it is common that brides are advised by their parents to carefully hide conflict and to protect the husband's honor from people outside the family. Accordingly, in our study, we found that the powerful self of these women, what Patzel (19) calls *self-efficacy*, was counteracted by rural isolation and cultural values that emphasized family unity (43). The women in our study were thus faced with an internal conflict that hampered their ability to optimally utilize their powerful self. Therefore, this inner conflict is an important internal factor that must be taken into account in the scheme of women's empowerment efforts, in terms of enhancing their coping skills.

Overall, the coping responses described by Moos (26) and Tobin et al. (27) resemble the coping strategies used by the women in this study. They used different coping strategies that changed over time, repeatedly oscillating between avoidance or disengagement to approaching or engaging. This fits with the findings of Waldrop and Resick (23), who claim that within an abusive relationship, a woman may prefer to use a certain coping strategy, but then she must adjust that strategy to fit to a particular situation. This is because women who experience domestic violence most likely face a lack of available resources as well as social support, options for escape, and control (44). The use of approach or engagement coping responses to counter the abuse led some of the women to be in a strong position to take action to leave the abusive husband, but, later on, that strategy also led them to face retaliation from their husband. Goodman et al. (44) have shown that active coping actions (approach and engagement responses) have the potential to positively affect the elimination of violence in a woman's life but at the same time have negative consequences. This is similar to what we found in our study. When women actively sought institutional support from the police, it stopped their husband's physical abuse but also led to other psychosocial problems such as a lack of financial support for the household. The dynamics of the abusive relationship and the overall availability of internal and external resources formed an elastic band strategy among the women survivors of domestic violence. This coping strategy will lead to further decisions about whether to stay or to leave the marriage.

### Validity

Using a phenomenological approach means that the researcher builds on people's experiences of a certain phenomenon to become more experienced themselves (39). The need for validating the findings by discussing the transcripts with the informants themselves is strongly emphasized (45, 46) and is similar to what Lincoln and Guba (47) call member checking. In this study, it was only possible to perform member checking with two of the informants. However, the prolonged engagement by the first author in the field of domestic violence (being a psychologist) as well as her frequent field visits to the study site increases the validity of the study. During the analysis, regular peer debriefing sessions were held within the research group to broaden the perspective and discuss the interpretation of the data.

### Limitations of the study

The study limitations refer mainly to the sampling of informants. All the interviewed women had accessed institutional services and they all had more than 9 years of education. This means that we do not know if the described coping mechanisms apply also to less educated women who have not sought support. These groups may be more prone to accept their situation and remain in an abusive relationship.

## Conclusion and policy implications

In this setting, domestic violence is still seen as a private and internal issue in which people outside the family are not supposed to intervene. Therefore women survivors of violence face a lack of institutional support and tend to have traditional beliefs that hamper their potential to stop the abuse. The women in our study had a fairly high educational level and had their own jobs; yet, they had difficulties in mobilizing the support needed to end the abuse. This could partly be explained by internalized gender norms about their responsibility for maintaining family harmony.

Women in this rural area face many disadvantages in life, including experiencing domestic violence, which is partly accepted by patriarchal gender norms. We encourage the government of Purworejo to:

- Conduct early prevention efforts through various public educational strategies to challenge the traditional norms, gender equity, and the unfair burden of gender-based violence in the community, among men and women, boys and girls.
- Establish a responsive law enforcement system so that all women survivors’ reports are followed up appropriately along with the Indonesia Domestic Violence Act (Law number 23/2004).
- Provide consistent resources such as funds for the daily management of the service unit and capacity building for the staff. This effort will guarantee the existence of the service unit in providing any service for women survivors of violence in the district.
- Improve their outreach service programs for women in remote areas by providing trained lay counselors at the community level. These lay counselors will be acting as the first care taker for further referral and ‘the first aid’ provider for women survivors before they undergo further referral to the unit.

Finally, the Government of Purworejo must take into account the specific needs of its inhabitants and understand that maintaining family harmony at any cost may threaten the lives of women and children in their area.
